# *Facklamia hominis* bacteremia after transurethral resection of the prostate: a case report

**DOI:** 10.1186/s12894-020-00762-8

**Published:** 2020-12-07

**Authors:** Miriam Gahl, Thomas Stöckli, René Fahrner

**Affiliations:** 1 Department of General, Visceral and Thoracic Surgery, Bürgerspital Solothurn, Schöngrünstrasse 42, 4500 Solothurn, Switzerland; 2 Department of Internal Medicine, Bürgerspital Solothurn, Schöngrünstrasse 42, 4500 Solothurn, Switzerland

**Keywords:** Bacteremia, *Facklamia hominis*, Transurethral resection of the prostate, Genital flora

## Abstract

**Background:**

Transurethral resection of the prostate (TUR-P) is one of the most frequent routine procedures in urology. Because of the semisterile environment, postoperative infections, including sepsis, are a common complication, with *Escherichia coli*, *Klebsiella* spp., *Proteus mirabilis* or *Enterococcus faecalis* as frequently isolated pathogens. *Facklamia hominis* is a gram-positive, facultatively anaerobic, alpha-hemolytic, catalase-negative coccus that was first described in 1997. To date, only a few cases of infectious complications have been described. We report the first case of postoperative bacteremia due to *Facklamia hominis* after TUR-P.

**Case presentation:**

An 82-year-old man developed fever only a few hours after elective TUR-P because of benign prostate syndrome. After cultivation of blood cultures, antibiotic therapy with ceftriaxone was intravenously administered and changed to oral cotrimoxazole before discharge of the afebrile patient. One anaerobic blood culture revealed *Facklamia hominis*. Under antibiotic therapy, the patient remained afebrile and showed no signs of infections during follow-up.

**Conclusions:**

Fever and bacteremia are frequent complications after TUR-P. This study is the first report of *Facklamia hominis* in a postoperative blood culture after TUR-P. To date, there are only a few reports of patients with infectious complications and isolation of *Facklamia hominis* in various patient samples. Because *Facklamia hominis* resembles viridans streptococci on blood agar analysis, this pathogen may often be misidentified. In this case identification of *Facklamia hominis* was possible with matrix-assisted laser desorption/ionization time-of-flight mass spectrometry. It has been postulated that *Facklamia hominis* might be a facultative pathogen and that its incidence will increase in the future.

## Background

Transurethral resection of the prostate (TUR-P) is one of the most frequent routine procedures in urology. Because of the semisterile environment and continuous rinsing with water, postoperative infections, including sepsis, are a common complication [1, 2]. Despite this fact, prophylactic antibiotics are still controversial, and applied substances vary in regard to the local spectrum of bacteria [1–4]. As a sign of procedural bacteremia, postoperative fever is often encountered early after surgical intervention. The most frequently isolated pathogens are *Escherichia coli*, *Klebsiella* spp., *Proteus mirabilis* and *Enterococcus faecalis*, which are also often detected during simple cystitis [5]. To cover these pathogens, preoperative prophylaxis with a single dose of oral ciprofloxacin is usually given in our department. In a case of postoperative urinary tract infection, daily ceftriaxone is administered intravenously, with a change to oral treatment before discharge. The antibiotic prophylaxis regarding the guidelines of the European Association of Urology should include the local pathogen prevalence and might thus differ from center to center [6].

To the best of our knowledge, we present the first case of *Facklamia hominis* bacteremia during the postoperative course after urological surgery.

## Case presentation

An 82-year-old man underwent elective TUR-P because of a symptomatic benign prostate syndrome. Preoperatively, no urinary sample was analyzed regarding bacterial colonization. He had a past history of cerebrovascular insult with minimal residuals, curative surgery for an adenocarcinoma of the rectum and cervical discus hernia. In addition, he suffered from hypertensive cardiopathy with a normal ejection fraction.

One hour after an uneventful operation, he developed chills that were successfully treated with pethidine. Three hours later, he developed a fever up to 38.7 °C so that two pairs of blood cultures were taken before initiating intravenous antibiotic therapy with ceftriaxone. Because of postoperative continuous rinsing of the bladder, it was impossible to cultivate the urine. The further course was uneventful, the patient remained afebrile and was in good condition so that the antibiotic therapy was changed to oral cotrimoxazole, and the patient was discharged. To our surprise, one out of four blood cultures turned positive for *Facklamia hominis* after the discharge of the patient. As the patient remained afebrile and in good clinical condition under the current antibiotic treatment, the therapy was continued for 14 days, although cotrimoxazole has not been described as a therapy so far. During 6 months of follow-up, the patient did not develop fever or signs of an urinary tract infection and had no need for antibiotic therapy again. During follow-up, there were urine and blood cultures without detection of *Facklamia hominis*.

## Discussion and conclusions

In our case, bacteremia with *Facklamia hominis* was detected in blood cultures of a patient after TUR-P only a few hours after the intervention. *Facklamia hominis* is a gram-positive, facultative anaerobic, alpha-hemolytic, catalase-negative coccus [7]. It was first described in 1997 [8], and for six clinical isolates, previously nonclassified cocci were characterized by phenotypic and phylogenetic methods as *Facklamia hominis* [9]. Since then, *Facklamia hominis* has been isolated in several specimens, such as urine, vaginal swabs, abscesses, joints, mitral valves, placentas, gastric aspirates, cerebrospinal fluid, preputial swabs, and blood [10–15]. Moreover, *Facklamia hominis* is thought to be a resident of the bacterial flora of the vaginal and urinary tracts [14]. Interestingly, *Facklamia hominis* has been so far isolated predominantly from females. In total, 16 cases of *Facklamia hominis* infections have been reported worldwide (Table 1). Furthermore, five other species of the genus Facklamia have been described, namely, *Facklamia ignava* [17], *Facklamia sourekii* [18], *Facklamia languida* [19], *Facklamia miroungii* [20] and *Facklamia tabaciasalis* [21]. Except for *Facklamia tabaciasalis* [21] and *Facklamia miroungii* [20], all Facklamia species have been isolated from human clinical specimens. It is postulated that, as *Facklamia* spp. resemble viridans streptococci on 5% sheep blood agar, they might have been confounded in the past with this group of organisms [9, 16].

**Table 1:** Overview of *Facklamia hominis* infections in the current literature

Author	Year	Number of patients	Type of sample	Outcome
Collins [8]	1997	6	Urine, vaginal swab, blood, abscess	Not reported
Healy [10]	2005	2	Blood, placental tissue, gastric aspirate	Cured
Safavi [11]	2010	1	Blood	Died
Ananthakrishna [16]	2012	1	Blood	Cured
Corona [7]	2014	1	Joint	Cured
Parvataneni [9]	2015	1	Cerebrospinal fluid	Cured
Abat [15]	2016	1	Abscess	Not reported
Schlipkoter [12]	2017	1	Abscess	Cured
Gomez-Luque [13]	2019	1	Preputial swab	Cured
Mostafa [14]	2019	1	Urine	Cured

This study is the first case reported with *Facklamia hominis* bacteremia after TUR-P. There have been reports about isolations from patients with abscesses, joint infections, endocarditis with positive blood cultures, cerebrospinal fluid, urine and vaginal swabs (Table 1). It has been postulated that *Facklamia hominis* might be a resident of the vaginal and urinary tract floras and a facultative pathogen inducing urinary tract infections [14]. In the reported case, the source of *Facklamia hominis* is speculative and might be displaced during surgery from the urinary tract or urine. Furthermore, the prostate might be colonized, but microscopy of the surgical tissue failed to detect large amount of bacteria. Fever or infections after TUR-P are frequently seen complications, as the intervention is semisterile, and microorganisms located within the urinary tract are often opportunistic. These facultative pathogens are common sources of postoperative bacteremia or urinary tract infections [1, 2]. Accordingly, it is not surprising that *Facklamia hominis* was now isolated in blood cultures after TUR-P. The treatment includes immediate antibiotic therapy depending on the prevalent resistance pattern after cultivation of blood and urine. To obtain an optimal antibiotic therapy, the isolation of the underlying pathogens is mandatory.

Traditional microbiological methods are often ineffective in correctly detecting pathogens such as *Facklamia hominis* [15]. Current methods of identification include matrix-assisted laser desorption/ionization time-of-flight mass spectrometry (MALDI-TOF) [13, 15], genome sequencing [14], rRNA PCR [7], VITEK 2 system [9, 16] and characterization by the API Rapid ID32 and API ZYM method [8]. Comparable to previous reports, in our case, *Facklamia hominis* was detected by MALDI-TOF.

Several treatment regimens have been described so far. They include penicillin derivatives, beta-lactamase inhibitors, metronidazole, cephalosporins, carbapenems, aminoglycosides, and glycopeptide antibiotics [7, 10–12, 16]. In our case, the intravenous treatment with ceftriaxone for 3 days and cotrimoxazole for a total of 14 days was successful. Whether the intravenous administration of ceftriaxone alone would have been sufficient as treatment is unclear. However, the patient rapidly recovered and remained afebrile without signs of bloodstream or urinary tract infection.

It is possible that due to the morphologic resemblance to viridans streptococci and ineffectiveness of traditional microbiological testing, *Facklamia hominis* has probably been often misdiagnosed in the past [15]. Whether *Facklamia hominis* will be an emerging pathogen in the future needs to be confirmed, but additional reports on antibiotic therapy are needed.

## Data Availability

The datasets used and/or analyzed during the current study are available from the corresponding author on reasonable request.

## Abbreviations

C: Celcius
MALDI-TOF: Matrix-assisted laser desorption/ionization time-of-flight mass spectrometry
spp.: species
TUR-P: Transurethral resection of the prostate
